# Impact of intracardiac pattern matching settings on the activation map of accessory pathways using open‐window mapping

**DOI:** 10.1002/joa3.70036

**Published:** 2025-03-09

**Authors:** Tomoyoshi Morioku, Yasuyuki Egami, Yasuharu Matsunaga‐Lee, Masamichi Yano, Masami Nishino

**Affiliations:** ^1^ Osaka Rosai Hospital Osaka Japan; ^2^ Division of Cardiology Osaka Rosai Hospital Osaka Japan

**Keywords:** accessary pathway, activation map, intracardiac pattern matching, open window mapping

## Abstract

This case report demonstrates that the ICPM‐A/V setting in open window mapping reduces misannotations and improves mapping accuracy for accessory pathways.

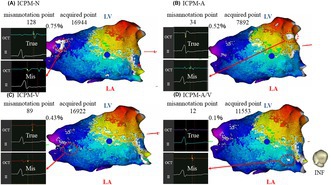

A 27‐year‐old male patient presented with symptomatic narrow QRS tachycardia and underwent an electrophysiological study (EPS) and catheter ablation. The tachycardia was diagnosed as orthodromic atrioventricular reentrant tachycardia (AVRT) via a left posterior accessory pathway (AP). Conventional AP mapping involves a point‐by‐point evaluation of local electrograms and detailed observation of atrial or ventricular potentials during retrograde or antegrade conduction. This process is often difficult and time‐consuming. Recently, open‐window mapping (OWM) with a 3D‐navigation system has emerged as a novel technique for the localization and ablation of APs.^1^ The intracardiac pattern matching (ICPM) algorithm, integrated within the CARTO3 system version 7.2 (Biosense Webster, Diamond Bar, CA, USA), The ICPM software uses 10 unipolar signals as references to detect changes, assigning patterns to maps based on correlation scores. Normalized correlation is calculated per channel using reference and arrhythmia waveforms, with scores derived from a weighted sum of each channel's results. By setting ICPM, the effects of ectopic beats induced by catheter stimulation can be eliminated, leading to more accurate mapping.^2^ By combining the ICPM algorithm with the Confidense™ module—which includes automatic mapping, wavefront annotation, and pattern matching—precise cardiac activation delineation is expected. This study examined the impact of ICPM settings on AP activation maps created using OWM. Activation maps were generated with the CARTO3 system and OCTARAY catheter (Biosense Webster) during tachycardia, using the maximum atrial potential from the distal coronary sinus (CS) as a reference. Acquired points and misannotations were analyzed offline under four ICPM settings:

1. ICPM‐N: No ICPM.
2. ICPM‐A: 10 unipolar atrial potentials from CS electrodes.
3. ICPM‐V: 10 unipolar ventricular potentials from CS electrodes.
4. ICPM‐A/V: 10 unipolar atrial and ventricular potentials from CS electrodes.

Since it has been reported that the early‐meets‐late (EML) algorithm can be used to estimate the valve annulus,^3^ in this case report, the EML threshold was set at 30% to define the atrioventricular border, with the valve annulus shown as a white line. Misannotations were defined as white lines deviating over 1 cm from the EML‐identified annulus, and their frequency was recorded. The settings of the activation map were as follows: cycle length stability of 5%, ICPM threshold of 0.75, positional stability of 3 mm, local activation time (LAT) stability of 3 mm, and maximum density. The range of the window of interest (WOI) was set to include atrial and ventricular potentials. The range of the pattern of interest (POI) was defined as follows for each ICPM setting:

- ICPM‐A: From −10 ms to +85 ms (Figure 1A).
- ICPM‐V: From −100 ms to −10 ms (Figure 1B).
- ICPM‐A/V: From −100 ms to +85 ms (Figure 1C).

**FIGURE 1:**
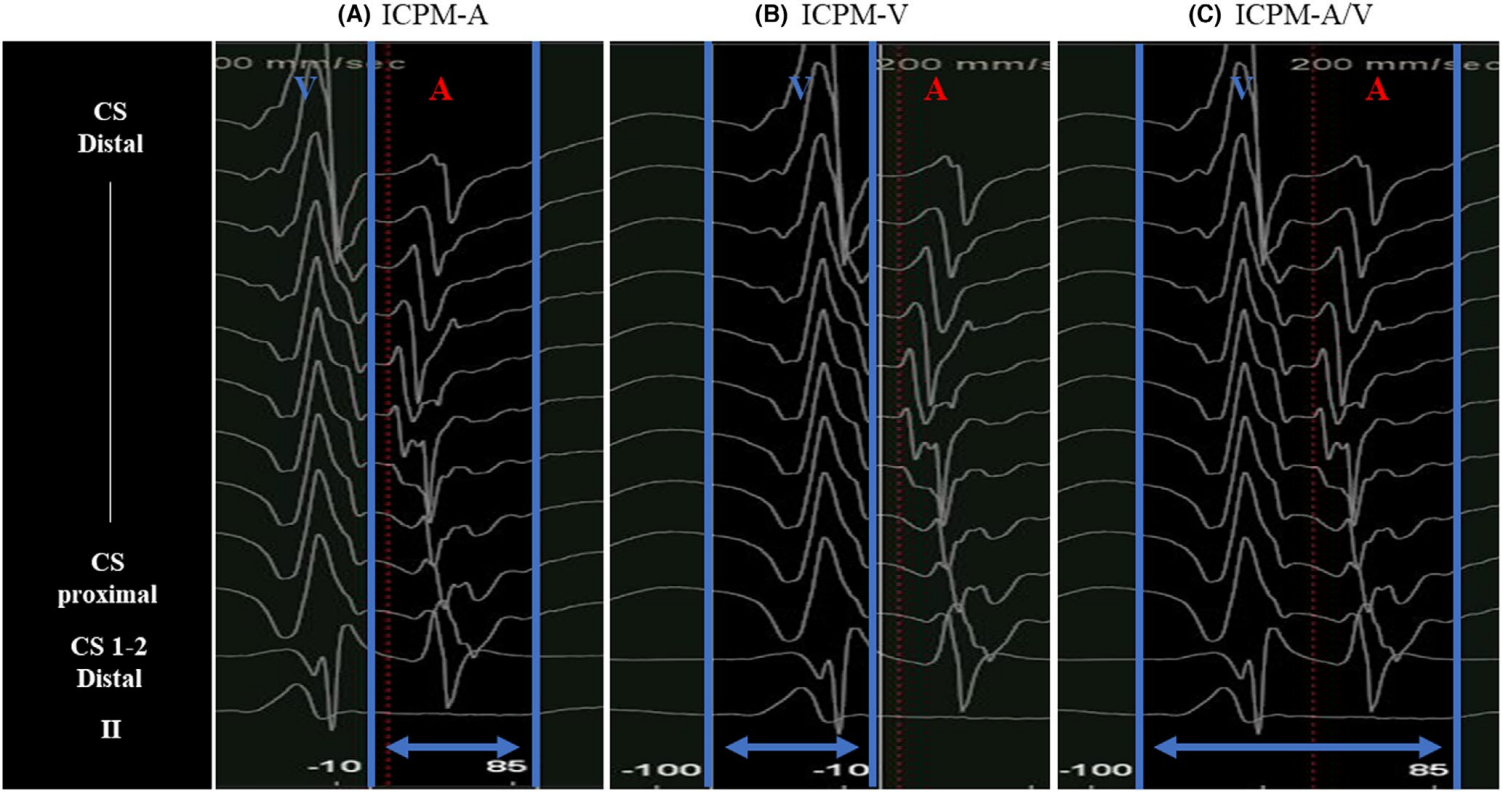
The range of POI settings for ICPM‐A (A), ICPM‐V (B), and ICPM‐A/V (C) during AVRT. AVRT, atrioventricular reentrant tachycardia; ICPM, intracardiac pattern matching; POI, pattern of interest.

The number of acquired points was as follows:

- ICPM‐N: 16 944 points (Figure 2A).
- ICPM‐A: 7892 points (Figure 2B).
- ICPM‐V: 16 922 points (Figure 2C).
- ICPM‐A/V: 11 553 points (Figure 2D).

**FIGURE 2:**
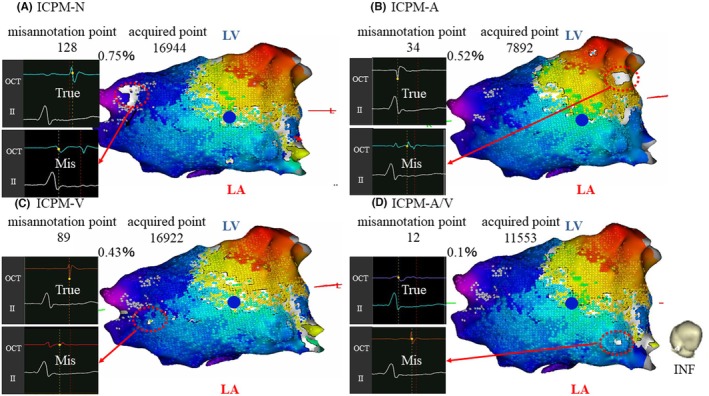
The number of acquired points under ICPM‐N settings (no use of ICPM) (A), ICPM‐A settings (B), ICPM‐V settings (C), and ICPM‐A/V settings (D). The red‐dotted circles indicate the local potentials of misannotations for each ICPM setting. The blue circle indicates the successful ablation site. A, atrium; A/V, atrium/ventricle; LA, left atrium; LV, left ventricle; V, ventricle.

Among these settings, ICPM‐A yielded the lowest number of acquired points. Misannotations were observed in the activation maps generated using ICPM‐N, ICPM‐A, and ICPM‐V settings. The misannotation data for each setting are as follows: In ICPM‐N, 128 misannotations were observed out of 16 944 points, accounting for 0.75% (Figure 2A). In ICPM‐A, 34 misannotations were identified out of 7892 points (0.52%) (Figure 2B). In ICPM‐V, 89 misannotations occurred out of 16 922 points (0.43%) (Figure 2C). Finally, in ICPM‐A/V, 12 misannotations were found out of 16 922 points (0.1%) (Figure 2D). ICPM‐A/V had the lowest frequency of misannotations among all settings. The ICPM algorithm enhances the identification of subtle changes in atrial and ventricular potentials by using multiple unipolar electrograms as references, thereby minimizing the acquisition of nontarget rhythms. In the present case, the ICPM‐A activation map had the fewest acquired points owing to excluding points affected by atrial potential polarity changes from reference catheter movement during respiration and cardiac motion. Additionally, 34 misannotations were observed, 29 of which were in ventricular regions (marked as ①), where ventricular potentials were mislabeled as atrial potentials, creating a white line on the map (Figure 3A). The red circle in Figure 3A shows ventricular sequence changes without atrial changes, but the ICPM score of 91 met the threshold, causing misannotations (Figure 3B). ICPM‐A's focus on atrial potentials, not ventricular, led to frequent ventricular misannotations. Similarly, the ICPM‐V map had 89 misannotations, 72 in atrial regions (marked as ②), where atrial potentials were mislabeled as ventricular because of detected atrial changes without ventricular changes (Figure 3C). The ICPM score of 87 met the threshold, causing misannotations (Figure 3D). ICPM‐V's focus on ventricular potentials caused frequent atrial misannotations. In contrast, ICPM‐A/V, monitoring both atrial and ventricular potentials, had the fewest misannotations, improving mapping accuracy. In the present case, the misannotation frequency with ICPM‐A/V was reduced from 0.75% to 0.1% compared to ICPM‐N, representing an 86.7% reduction, leading to the creation of a more accurate activation map. This improvement may enhance ablation success rates; furthermore, by avoiding unnecessary ablations, it is expected to reduce complications related to the procedure. In fact, in this case, AP conduction was eliminated within 2 s of the first radiofrequency application using ICPM‐A/V mapping, with no recurrence during the 1‐year follow‐up. Similarly, in another case (Data S1), AP conduction was eliminated within 3 s using the same method, and neither case showed recurrence. ICPM‐A/V provided more accurate activation maps, potentially reducing ablation time. Additionally, the time required to configure ICPM‐A/V is almost negligible compared to conventional mapping, making it a feasible option for routine clinical use. Furthermore, ICPM‐A/V demonstrated applicability to diverse accessory pathways, as seen in this case (left posterior AP) and File S1 (right lateral AP). However, since this is a case report, further investigation involving a larger number of cases will be necessary to validate its clinical utility in the future. In addition, the Local Velocity Vectors module in CARTO3 version 8 visualizes wave propagation using arrows, simplifying activation patterns and aiding in diagnostics. With ICPM‐A/V, accurate annotations emphasize AP conduction pathways through directional arrows, while clearer identification of annular regions (without arrows) may improve ablation success rates.

**FIGURE 3:**
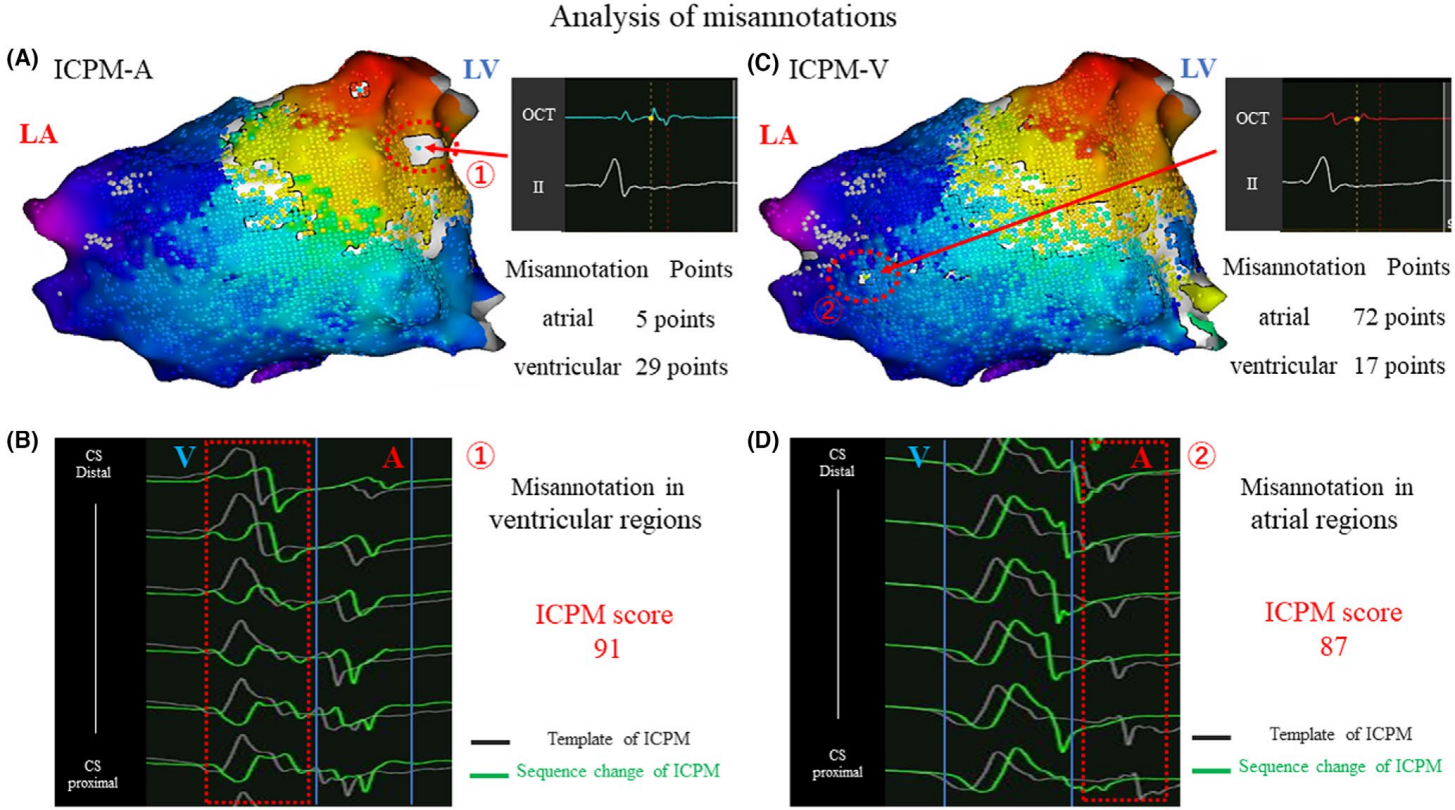
Analysis of misannotations: (A) Misannotations in the ventricular region for ICPM‐A. (B) Unipolar potentials showing misannotation in ICPM‐A. (C) Misannotations in the atrial region for ICPM‐V. (D) Unipolar potentials show misannotation in ICPM‐V.

## CONFLICT OF INTEREST STATEMENT

The authors declare that there is no conflict of interest.

## CONSENT

The patient provided written informed consent for the ablation procedures and agreed to the publication of his case details and images in this report.

## Supporting information


Data S1.

## Data Availability

The data that support the findings of this study are available from the corresponding author upon reasonable request.
