# Supplementary figures and images for: Impact of intracardiac pattern matching settings on the activation map of accessory pathways using open‐window mapping
Source: J Arrhythm. 2025 Mar 9;41(2):e70036. doi: 10.1002/joa3.70036 (PMC11891396; doi:10.1002/joa3.70036)

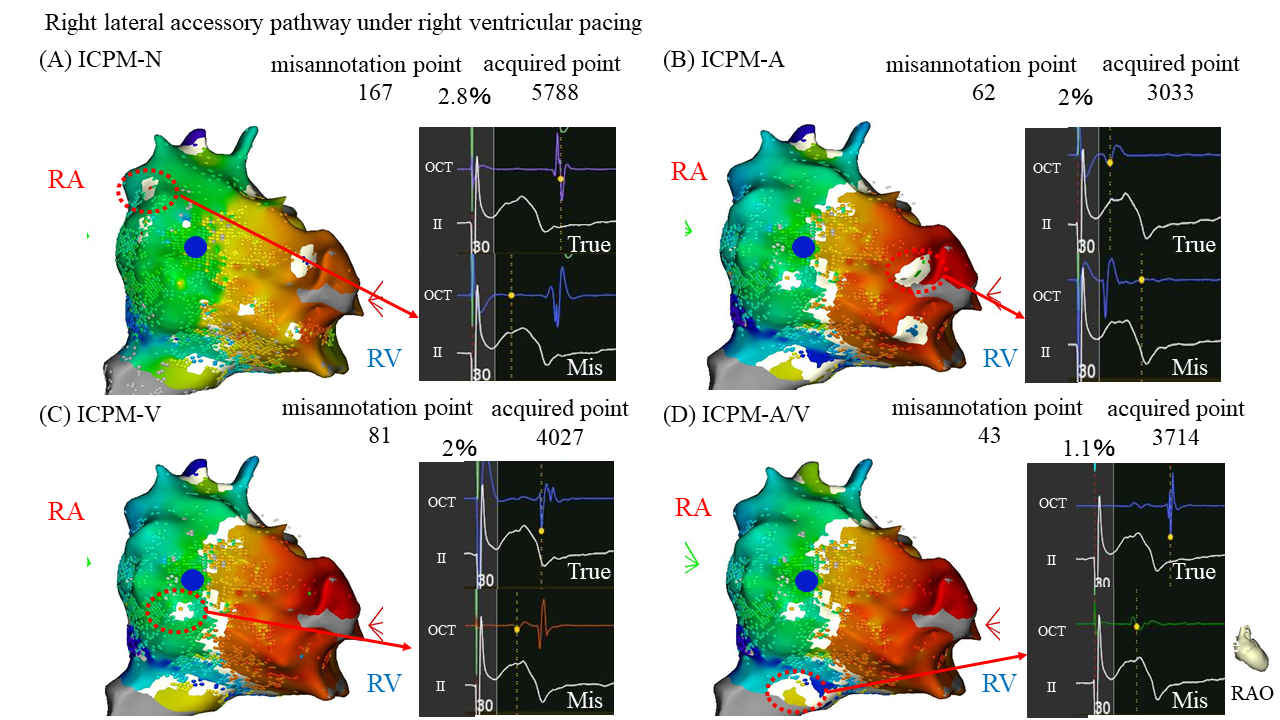

Supplement: Supplementary file 1 — Data S1. [file JOA3-41-e70036-s001.tif]
